# Computed tomography-based navigation versus accelerometer-based portable navigation in total hip arthroplasty for dysplastic hip osteoarthritis

**DOI:** 10.1007/s00590-025-04188-6

**Published:** 2025-03-01

**Authors:** Shinya Tanaka, Yusuke Osawa, Yasuhiko Takegami, Hiroto Funahashi, Hiroaki Ido, Takamune Asamoto, Shiro Imagama

**Affiliations:** Department of Orthopaedic Surgery, Nagoya University Graduate School of Medicine, Nagoya-shi, Japan

**Keywords:** Acetabular cup orientation, Hip osteoarthritis, Computer-assisted surgery, CT-based navigation system, Portable imageless navigation system, Total hip arthroplasty

## Abstract

**Purpose:**

Accurate cup placement is challenging in total hip arthroplasty (THA) for dysplastic hip osteoarthritis (DHOA) because of the complex morphology of the acetabulum. Studies have reported good accuracy for total hip arthroplasty (THA) using computed tomography-based navigation (CTN); however, in recent years, portable navigation (PN) has become more widely applied because of its low cost and ease of use. This study aimed to compare the accuracy of portable navigation with that of CT-based navigation.

**Methods:**

A total of 114 patients underwent THA for DHOA via the standard posterior approach in the lateral decubitus position using the CTN (CTN-THA group) or PN (PN-THA group) system. After propensity score matching, 32 patients were included in each group. The accuracy of cup inclination, anteversion, cup placement position, and operative time were compared between the groups.

**Results:**

There was no difference in accuracy error between the CTN-THA (inclination 2.8 ± 2.0° and anteversion 3.4 ± 2.1°) and PN-THA groups (inclination 2.5 ± 1.8° and anteversion 2.6 ± 2.2°). The CTN-THA group (inclination 2.2 ± 2.0° and anteversion 2.1 ± 1.6°) achieved better navigation error compared to the PN-THA group (inclination 2.6 ± 2.2° and anteversion 3.8 ± 3.3°). The error of cup placement position in the anteroposterior direction was significantly larger in the PN-THA group (4.27 ± 3.02 mm) than in the CTN-THA group (2.13 ± 2.17 mm). The operative time was significantly longer in the CTN-THA group (115 ± 41 min) than in the PN-THA group (87 ± 19 min).

**Conclusions:**

CTN-THA exhibited better accuracy than PN-THA for both cup placement angles and positions. CTN-THA tended to increase the operative time compared to PN-THA.

## Introduction

Approximately 80% of hip osteoarthritis (HOA) cases in Japan are due to acetabular dysplasia [1]. In total hip arthroplasty (THA) for dysplastic hip osteoarthritis (DHOA), accurate cup placement is more challenging than in primary osteoarthritis due to complex acetabular morphology [2]. This accuracy is crucial to avoid complications such as dislocation, impingement, and polyethylene wear [3, 4]. Dislocation is a leading cause of revision and has become a significant challenge for hip surgeons as implant longevity improves. Various computer-assisted surgery (CAS) systems have been developed to enhance cup placement accuracy [5].

Computed tomography (CT)-based navigation employs a CAS system. Currently, surface matching is the primary registration method, allowing confirmation of accuracy by checking landmark positions post-registration. Previous studies indicate that CT-based navigational THA (CTN-THA) outperforms conventional THA in cup placement position and angle [6]. However, CTN-THA often results in longer operative times, which can increase blood loss, readmissions, reoperations, and wound infection rates [7].

Portable navigation (PN) has been widely used in recent years owing to its low cost and ease of use. Currently, available PNs can be registered using two main methods: lateral position registration, which is based on the direction of the body axis, and supine position registration, which uses the functional pelvic plane (FPP) as a reference, based on both the anterior superior iliac spines and gravity axis [8–10]. Registration in the lateral position is generally considered inaccurate because it is affected by the pelvis position at the time of lateral fixation [11, 12]. The ‘flip technique’ has been reported as a more accurate method of placing the cup in the lateral position in THA, in which registration is performed in the supine position and then repositioned to the lateral position for surgery [10]. However, there are no reports evaluating the accuracy of cup placement in PN-THA using the ‘flip technique’ and CTN-THA.

The study aims to address the following clinical questions: (1) Is the cup placement accuracy of the PN-THA using the ‘flip technique’ for DHOA equivalent to that of the CN-THA?, (2) is the cup placement position of PN-THA using the ‘flip technique’ for DHOA equivalent to that of CN-THA?, (3) does PN-THA shorten the operative time compared with CTN-THA?

## Materials and methods

### Patients

**Fig. 1:**
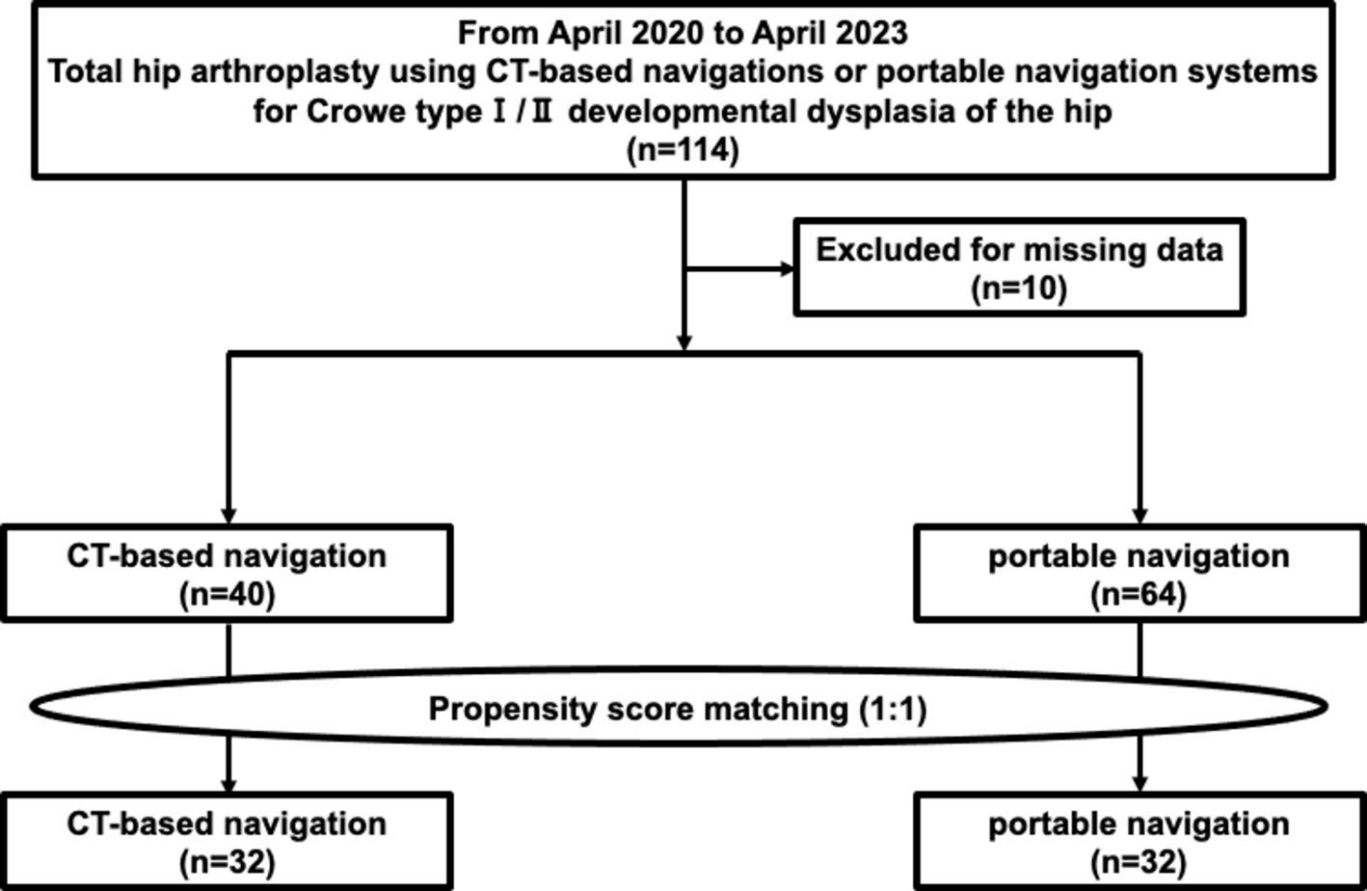
Flowchart of patient inclusion

The study was approved by our institution’s ethics committee and performed in line with the Declaration of Helsinki. All patients provided informed consent for participation and publication. Participants underwent THA with CTN or PN for DHOA (Crowe types I and II) at our institution from April 2020 to April 2023. This study included 45 patients in the CTN-THA group and 69 in the PN-THA group. Ten patients with missing data were excluded, five from each group, resulting in 104 patients. Propensity score matching adjusted for differences in patient backgrounds. Scores were calculated using logistic regression with age, sex, BMI, and Crowe classification as variables. The scores were matched 1:1, resulting in 32 cases for each group (Fig. 1). After matching, no significant differences were noted in age, sex, BMI, or Crowe classification (Table 1).

**Table 1:** Preoperative patient background

	CTN-THA		PN-THA		*p *value
Age (y)	64.0 ± 7.9		66.3 ± 8.3		0.253
Sex (Male/Female)	5/27		6/26		1
BMI (kg/m^2^)	24.8 ± 3.6		24.7 ± 3.4		0.855
Crowe classification (I/II)	29/3		29/3		
Cup size (mm)	50.0 ± 2.5		50.0 ± 3.6		1
Number of screws	1.8 ± 0.7		1.8 ± 0.6		0.715
Acetabular component					1
Cementless	Trident	28	SQRUM	32	
	Trident II	4			
Femoral component					0.103
Cemented	Exeter	6	SC	2	
Cementless	Secur-Fit Advanced	26	Initia	30	
JOA score (Preoperative)					
Pain	19.7 ± 9.5		21.8 ± 7.7		0.335
ROM	15.2 ± 4.0		14.3 ± 3.0		0.35
Gait	12.6 ± 4.0		13.1 ± 4.0		0.6
ADL	12.8 ± 2.7		13.6 ± 2.1		0.184

Data are shown as mean ± standard deviation or number

BMI, body mass index; JOA, Japanese Orthopedic Association; ROM, range of motion; ADL, activities of daily living

### Surgical procedure

Preoperative planning utilized 3D templating software (ZedHip, Lexi, Tokyo, Japan). ZedHip is a 3D preoperative templating software that uses CT images. It allows for the creation of multi-planar reconstruction images based on CT data and enables 3D templating of the stem and cup. The stem that best fit the femoral shape was selected on the 3D template. Cups from the same manufacturer as the stems were positioned at the center of the anatomical hip. If the cup Center–Edge angle was greater than 0°, THA with a cementless cup was planned; if less than 0°, reconstruction with a cemented cup and bulk bone graft was planned. The navigation system was chosen from the same manufacturer as the implant. The proportion of cemented stems in each group did not differ significantly (Table 1).

All procedures were performed using the posterior approach in the lateral decubitus position by experienced hip surgeons under the supervision of a single surgeon. The target angle for cup placement was determined based on the FPP in the supine position, with a target of 40° for inclination and 20° for anteversion according to Murray’s definition [13]. The use of screws for cup fixation was at the surgeon’s discretion based on intraoperative findings. Postoperatively, full weight bearing was allowed, and gait training was conducted. One week after surgery, CT confirmed the postoperative placement angle.

### Navigation system

#### CT-based navigation

**Fig. 2:**
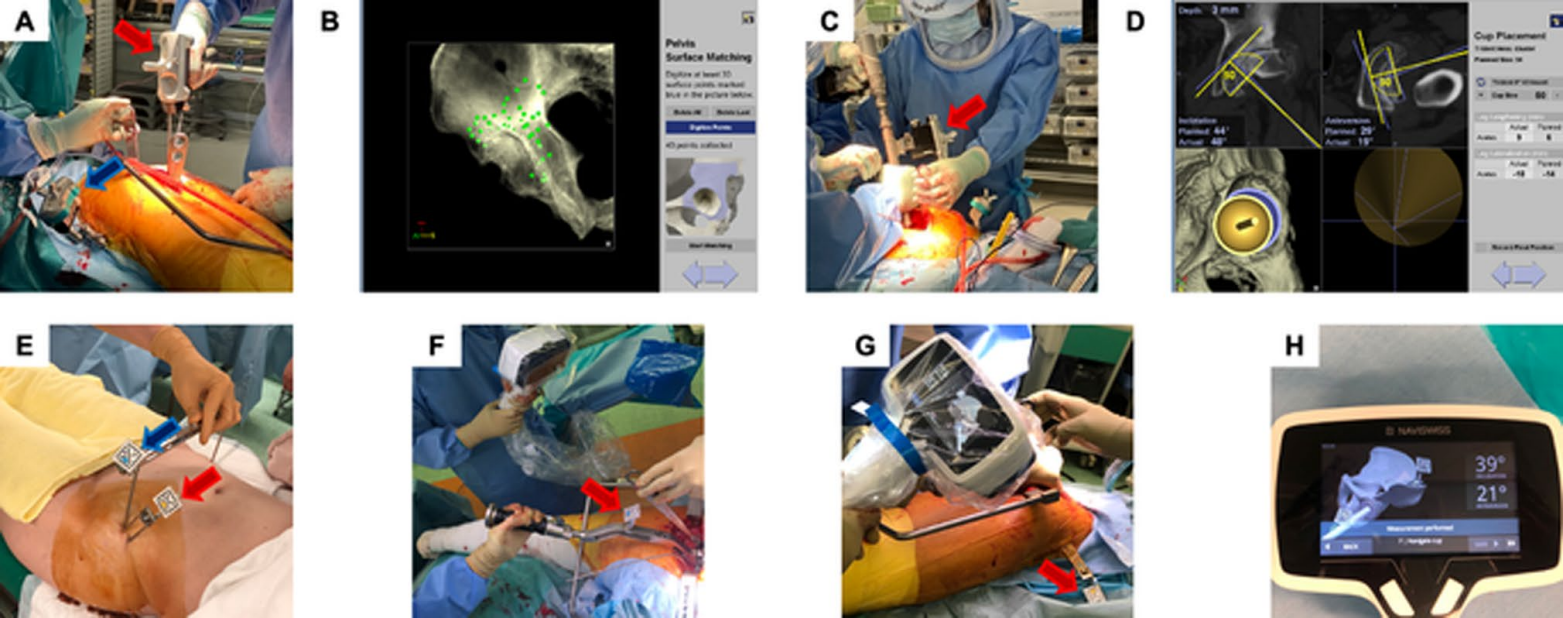
CT-based navigation system and portable navigation system. **A** CT-based navigation system. Intraoperative image during registration. The pelvic surface is palpated with a pointer (Indicated by the red arrow). The tracker is attached to the iliac crest (Indicated by the blue arrow). **B** Navigation screen during surface registration. **C** Navigation attached to the reamer (Indicated by the red arrow). **D** Navigation screen during cup placement. The angle and position of cup placement can be checked. **E** Portable navigation system. Registration is performed prior to surgery. The P-tag is attached to the pelvis (Indicated by the red arrow); both superior anterior iliac spines are palpated using a pelvic caliper with the M-tag (Indicated by the blue arrow). **F** The M-tag is attached to the cup holder (Indicated by the red arrow). **G** The P-tag is attached to the pelvis (Indicated by the red arrow). **H** Only the angle of cup placement can be checked intraoperatively

CT-based hip navigation system (Stryker, Mahwah, New Jersey, USA) was used for CTN. The pelvis was segmented, and coordinates were defined using several reference points. Before skin incision, two 4.0 mm diameter pins were inserted percutaneously into the iliac crest to which a pelvic tracker was attached (Fig. 2A). Intraoperatively, point-pair matching was initially attempted by registering the four representative points determined preoperatively. Surface matching was performed by registering > 30 points on the pelvic surface (Fig. 2B). The accuracy of the registration was verified by palpating representative landmarks using a pointer. Intraoperative navigation was utilized to verify the intended position and angle of the cup during reaming and cup placement (Fig. 2C, D) [14, 15].

#### Portable navigation

Naviswiss (Naviswiss AG, Brugg, Switzerland) is a system that uses an accelerometer with a small tag and navigation unit for PN. The navigation unit features an infrared stereo camera to measure the position and orientation of the tag, along with an inertial measurement unit with an accelerometer and gyroscope for spatial orientation. The ‘flip technique’ can be used with this system for THA in a lateral decubitus position. Registration is performed with the patient supine, followed by repositioning to the lateral position, keeping the pins and tags clean. Preoperatively, two 3.0 mm diameter pins are inserted percutaneously into the iliac crest, and a small tag (P-tag) is attached. The anterior superior iliac spines (ASISs) are palpated using a caliper with another small tag (M-tag) (Fig. 2E). Both tags are captured by an infrared camera to define the FPP based on the ASISs and the weight axis direction. During reaming and cup placement, the P-tag is attached to the pelvis (Fig. 2F), and the M-tag to the reamer or cup holder (Fig. 2G). A stereo camera reads these tags to verify the cup placement angle (Fig. 2H) [9, 10].

### Postoperative evaluations

Postoperative evaluation used 3D templating software (ZedHip). After importing CT images, the cup placement angle and position were measured by superimposing a template of the same size onto the actual cup [8]. The angle of cup placement was assessed for radiographic inclination (RI) and radiographic anteversion (RA) with reference to the FPP in the supine position. The accuracy error was calculated as the difference between the target angles (RI 40° and RA 20°) and the actual angle. Similarly, the navigation error was determined as the difference between the final navigation display value and the actual placement angle. Both values are expressed as absolute errors.

**Fig. 3:**
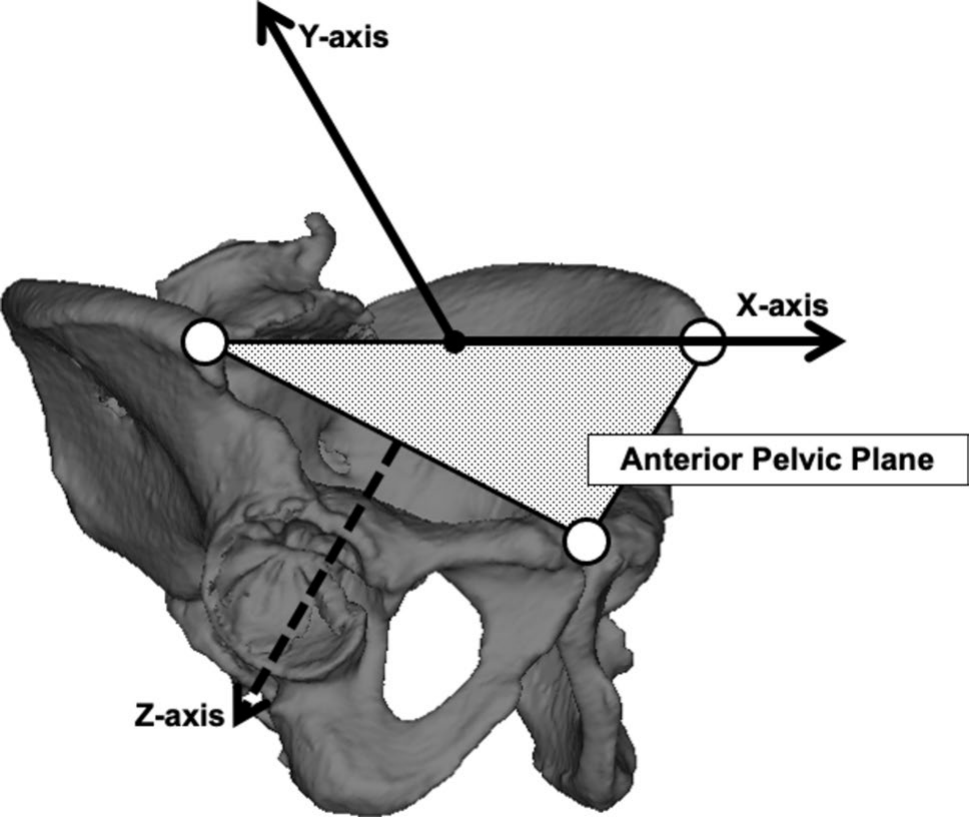
Three-dimensional coordinate system of the pelvis with reference to the anterior pelvic plane

**Fig. 4:**
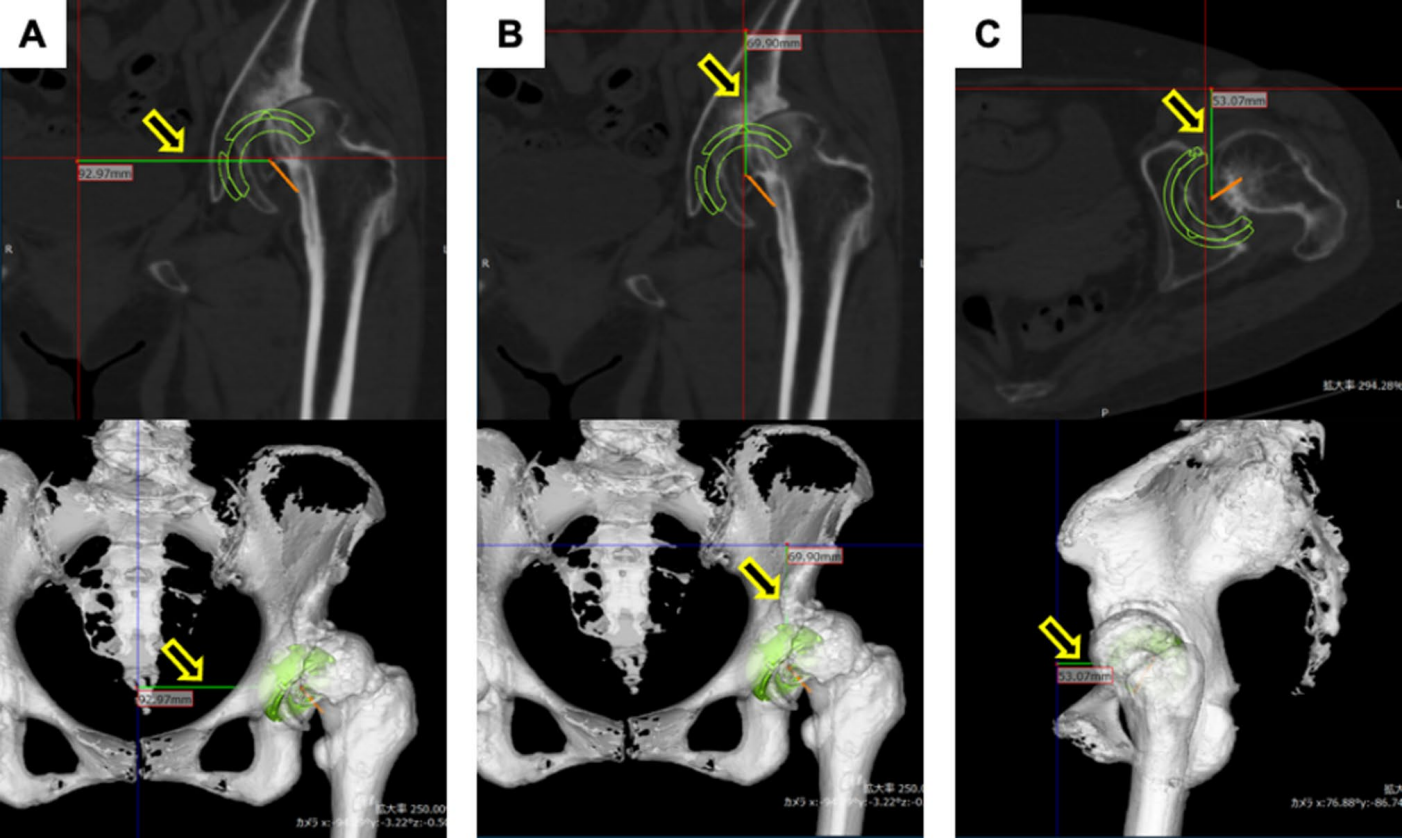
Measuring the cup center position using 3D templating software. The green line (indicated by the yellow arrow) was measured. **A** X-axis error (internal/external direction error). **B** Y-axis error (vertical direction error). **C** Z-axis (anteroposterior direction error)

To assess cup placement, a three-dimensional coordinate system was established with reference to the anterior pelvic plane (APP). The origin was defined as the midpoint between the ASISs. The X-axis represents a line through the bilateral ASISs. The Y-axis is parallel to the APP and passes through the origin. The Z-axis is perpendicular to the APP through the origin (Fig. 3). The cup’s center of rotation was measured before and after surgery using this coordinate system. The X-axis error indicates internal/external direction error, the Y-axis error indicates vertical direction error, and the Z-axis error indicates anteroposterior direction error. The cup center position recorded in ZedHip at preoperative planning was measured using the coordinate system (Fig. 4). Postoperative CT data were imported into ZedHip to measure the actual cup position. A template of the same size was superimposed on the actual placement position to evaluate the cup position along the X-, Y-, and Z-axes. Absolute errors between the actual cup position and the preoperative planned position on the X-, Y-, and Z-axes were evaluated [16].

### Surgical outcomes

We recorded the operative time and intraoperative and postoperative complications (dislocation, intraoperative fracture, surgical site infection, and nerve palsy). Additionally, clinical outcomes were evaluated using the Japanese Orthopedic Association (JOA) score at both preoperative and 1-year postoperative time points.

### Statistical Analysis

Based on previous reports, if a 3° navigation error was considered significant, 28 cases in each group would be required for a power of 0.8, with a significant difference of 0.05 [14]. Statistical analysis was conducted for each item using the *t*-test for continuous variables and Fisher’s exact probability test for nominal variables. *P* < 0.05 was considered significant. EZR version 1.65 (Saitama Medical Center, Jichi Medical University, Saitama, Japan) was used for statistical analysis.

## Results

**Fig. 5:**
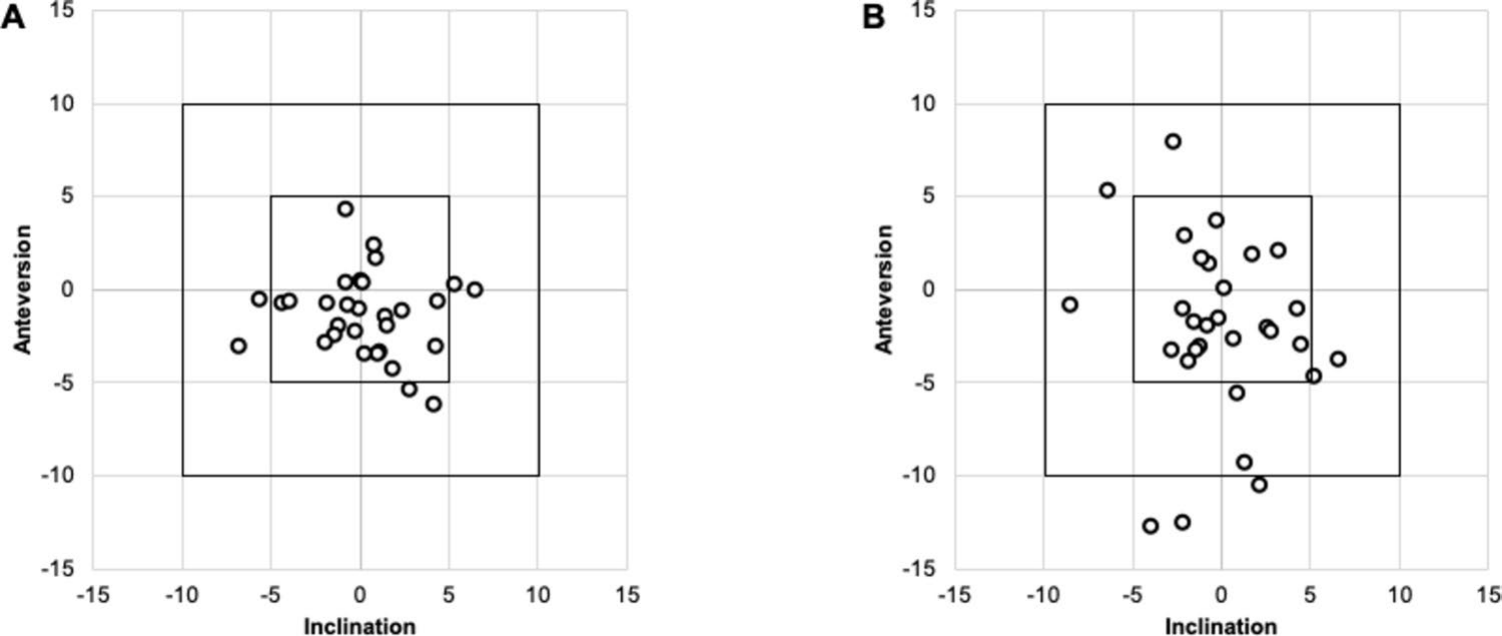
Scatter plot of navigation error. **A** CT-based navigation group. **B** portable navigation group

No significant differences were found in cup size, number of screws, or preoperative JOA scores between the groups (Table 1). For the CTN-THA group, cup placement angles were RI 39.8 ± 3.4° and RA 17.8 ± 3.5°; for the PN-THA group, RI 40.3 ± 3.1° and RA 18.3 ± 3.0°. The accuracy error for CTN-THA was 2.8 ± 2.0° in inclination and 3.4 ± 2.1° in anteversion; for PN-THA, it was 2.5 ± 1.8° in inclination and 2.6 ± 2.2° in anteversion. Navigation error for CTN-THA was 2.2 ± 2.0° in inclination and 2.1 ± 1.6° in anteversion, while for PN-THA, it was 2.6 ± 2.2° in inclination and 3.8 ± 3.3° in anteversion. The CTN-THA group had significantly smaller navigation errors than the PN-THA group (*p* < 0.01). The number of cases with navigation errors greater than 5° and 10° did not differ between groups (Fig. 5).

The error between planned and actual cup placement was 2.85 ± 1.79 mm on the X-axis, 2.47 ± 2.16 mm on the Y-axis, and 2.13 ± 2.17 mm on the Z-axis for CTN-THA. In the PN-THA group, the errors were 4.08 ± 3.23 mm in the X-axis, 3.62 ± 2.68 mm in the Y-axis, and 4.27 ± 3.02 mm in the Z-axis, with significantly smaller Z-axis (anteroposterior direction) errors in the CTN-THA group (*p* < 0.01; Table 2).

**Table 2:** Cup placement angles and positions

	CTN-THA	PN-THA	*p* value
Cup placement angles measured postoperatively			
Inclination (°)	39.8 ± 3.4	40.3 ± 3.1	
Anteversion (°)	17.8 ± 3.5	18.3 ± 3.0	
Cup placement angles displayed on the navigation screen			
Inclination (°)	39.5 ± 2.4	40.6 ± 2.1	
Anteversion (°)	19.0 ± 3.0	20.3 ± 3.0	
Absolute value of accuracy error ^a^			
Inclination (°)	2.8 ± 2.0	2.5 ± 1.8	0.591
Anteversion (°)	3.4 ± 2.1	2.6 ± 2.2	0.176
Absolute value of navigation errors ^b^			
Inclination (°)	2.2 ± 2.0	2.6 ± 2.2	0.486
Anteversion (°)	2.1 ± 1.6	3.8 ± 3.3	< 0.01
Outlier > 5	6	11	0.257
Inclination	4	5	1
Anteversion	2	7	0.148
Outlier > 10	0	3	0.238
Inclination	0	0	1
Anteversion	0	3	0.238
Cup placement position			
X-axis (mm)	2.85 ± 1.79	4.08 ± 3.23	0.06
Y-axis (mm)	2.47 ± 2.16	3.62 ± 2.68	0.06
Z-axis (mm)	2.13 ± 2.17	4.27 ± 3.02	< 0.01

Data are shown as mean ± standard deviation or number

^a^Absolute values of the differences between the angles measured postoperatively and the target angles

^b^Absolute values of the differences between the angles measured postoperatively and those displayed on the navigation screen

Operative time was significantly longer in the CTN-THA group (115 ± 41 min) compared to the PN-THA group (87 ± 19 min; *p* < 0.01). Intraoperative blood loss was similar (CTN-THA: 458 ± 276 mL, PN-THA: 384 ± 249 mL). No significant differences were found in complications or JOA scores one year post-surgery (Table 3).

**Table 3:** Surgical outcomes

	CTN-THA	PN-THA	*p *value
Operative time (min)	115 ± 41	87 ± 19	< 0.01
Intraoperative blood loss (mL)	458 ± 276	384 ± 249	0.358
Intraoperative complications	0	0	1
Postoperative complication	1	2	1
Infection	1	0	
Dislocation	0	1	
DVT	0	1	
JOA score (1 year after surgery)			
Pain	37.4 ± 7.5	38.4 ± 3.1	0.525
ROM	17.6 ± 2.1	17.7 ± 2.1	0.873
Gait	17.6 ± 3.3	17.7 ± 3.2	0.917
ADL	17.6 ± 2.3	16.3 ± 3.0	0.053
Total	90.2 ± 10.3	90.0 ± 7.2	0.91

Data are shown as mean ± standard deviation or number

DVT, deep vein thrombosis; JOA, Japanese Orthopedic Association; ROM, range of motion; ADL, activities of daily living

## Discussion

This study compares the accuracy of cup placement for DHOA between THA using PN with the ‘flip technique’ and THA using CTN. The results indicated that CTN-THA had a smaller navigation error in anteversion than PN-THA. In addition, CTN-THA showed fewer anteroposterior errors in cup positioning than PN-THA. The operative time was significantly shorter for PN-THA than for CTN-THA.

Generally, THA for DHOA is more challenging than primary HOA because of problems such as acetabular and femoral deformity, femoral head subluxation, and severe leg length differences. Specifically, placing the cup at the correct angle is difficult because of inadequate cup coverage due to a lack of bone stock, presence of a double floor compared with primary HOA [2]. CTN-THA has previously been reported to have good accuracy in cases with such a complex hip morphology and in obese patients [14, 17]. On the other hand, it has been reported that cup angle becomes inaccurate due to pelvic tilt during registration in the lateral position of PN-THA [11, 12]. To achieve more accurate cup placement in the lateral position of PN-THA, a registration method using the ‘flip technique’ was developed [10]. However, conventional imageless navigation with registration by palpation of both ASISs and the pubic symphysis has been shown to be less accurate in obese patients due to subcutaneous fat [18]. In our comparison, CTN-THA showed superior accuracy compared to PN-THA for both the angle and position of the cup. The difference in the accuracy between the two navigation systems can be attributed to the registration method. CTN performs registration by directly tapping the bone, and the accuracy of the registration can be confirmed by palpating representative landmarks. On the other hand, as with conventional imageless navigation, the thickness of subcutaneous fat may have affected the accuracy of registration for PN.

Compared to primary HOA, greater number of cases of DHOA involve anatomical abnormalities of the acetabulum. Placement of the cup in the anatomical hip center is difficult due to the difficulty in accurately identifying the location of the original acetabulum intraoperatively [19]. In conventional THA, the difference between preoperative planning and actual placement of the cup using a three-dimensional coordinate system is reported to be approximately 3–4 mm [20]. CTN-THA is known to have a more accurate cup position than conventional THA, with an error of approximately 1–2 mm [14, 16, 17]. However, there are no reports on cup placement in PN-THA. In our results, the error of the PN-THA was larger than that of the CTN-THA in the anteroposterior direction. While PN-THA can provide intraoperative information related to cup placement angles, it cannot provide information on cup placement positions. In contrast, CTN-THA provides information on the positions of the reamer and cup. These differences suggest that the accuracy of cup placement positions with CTN-THA is superior to that of PN-THA.

Studies have shown that CTN-THA may prolong the operative time compared with conventional THA [21]. However, the impact of PN on operative time is conflicting [22]. Our results showed that PN-THA required a significantly shorter operative time than CTN-THA. If the ‘flip technique’ is used, registration should be performed in the supine position before the skin incision [10]. Our results may have been affected by the fact that registration was performed before the start of surgery and by the simplicity of PN. PN has an advantage in terms of operative time, because longer operative times can lead to increased blood loss and risk of infection [7, 23].

This study has several limitations. First, the small number of cases may limit the detection of differences in clinical outcomes and complications, despite power analysis performed for placement angles. Second, the groups used different types of cups, potentially affecting placement position and angle due to fixation differences. Third, it is uncertain whether FPP is the same before and after THA. However, it is suggested that pelvic tilt after THA progresses over time, with minimal changes observed 1 year postoperatively [24, 25]. Considering this, since this study evaluates CT at 1 week postoperatively, the supine FPP before and after surgery is thought to be almost equivalent. Nonetheless, this study is the first to evaluate cup placement accuracy in PN-THA and CTN-THA for DHOA, which is more challenging than primary HOA. The findings will provide useful information for many hip surgeons.

In conclusion, the use of the ‘flip technique’ with the FPP as the reference plane in THA for DHOA resulted in significantly shorter operative time compared to CTN-THA. However, the CTN-THA demonstrated greater accuracy than the PN-THA for cup placement angles and positions, indicating the superiority of CTN.

## Data Availability

The datasets used and/or analyzed during the current study are available from the corresponding author on reasonable request.
